# Protective roles of free avian respiratory macrophages in captive birds

**DOI:** 10.1186/s40659-016-0090-7

**Published:** 2016-06-16

**Authors:** Mbuvi P. Mutua, Shadrack Muya, Muita M. Gicheru

**Affiliations:** Department of Zoological Sciences, Kenyatta University, P.O. Box 43844-00100, Nairobi, Kenya; Department of Zoology, Jomo Kenyatta University of Agriculture and Technology, P.O. Box 62000-00200, Nairobi, Kenya

**Keywords:** Avian lung, FARM, Phagocytosis, PPAR γ ligands

## Abstract

In the mammalian lung, respiratory macrophages provide front line defense against invading pathogens and particulate matter. In birds, respiratory macrophages are known as free avian respiratory macrophages (FARM) and a dearth of the cells in the avian lung has been purported to foreordain a weak first line of pulmonary defense, a condition associated with high mortality of domestic birds occasioned by respiratory inflictions. Avian pulmonary mechanisms including a three tiered aerodynamic filtration system, tight epithelial junctions and an efficient mucociliary escalator system have been known to supplement FARM protective roles. Current studies, however, report FARM to exhibit an exceptionally efficient phagocytic capacity and are effective in elimination of invading pathogens. In this review, we also report on effects of selective synthetic peroxisome proliferator activated receptor gamma (PPAR γ) agonists on non phlogistic phagocytic properties in the FARM. To develop effective therapeutic interventions targeting FARM in treatment and management of respiratory disease conditions in the poultry, further studies are required to fully understand the role of FARM in innate and adaptive immune responses.

## Introduction

In the mammalian lung, respiratory macrophages provide first line of defense where they expunge and remove particulate matter and kill invading pathogens [1]. The cells are resident and numerous on the luminal surface of alveoli, a strategic location that enables them to internalize and kill pathogens before breaking the epithelia barrier with a possibility of causing local and systemic infections [2]. While substantial data on the protective roles of mammalian respiratory macrophages is well documented [3, 4], little is known about the free avian respiratory macrophages (FARM) [5]. Despite different resident tissue macrophages being phenotypically and functionally distinct [4], some investigators have extrapolated data on avian blood monocytes, splenic macrophages and peritoneal macrophages to apply to the FARM [5].

The avian lung is susceptible to respiratory inflictions owing to its anatomical features that distinguish it from the mammalian lung. The avian blood-gas respiratory tissue barrier is 56–67 % thinner than that of a mammal of equivalent body weight and the respiratory surface is 15 % greater [6]. Skin inflictions predispose the avian respiratory system to airborne infections such as air sacculitis, a condition enhanced by some air sacs diverticulae that extend beyond the coelomic cavity and lie near the skin surface [7]. Additionally, among the vertebrates, birds have the most efficient respiratory system that extracts 60 % of inhaled oxygen compared to mammalian lung that extracts 27 % of oxygen in inspired air, predisposing the avian lung air sac system to oxidative stress [8]. The vast and attenuated avian blood-gas respiratory tissue barrier not only enhances flux of gases by diffusion, but also facilitates invasion of the lungs by particulates and pathogens [9]. Without development of an apt pulmonary defense system, birds would be more vulnerable to respiratory disease conditions.

## Lavage of avian lungs yields few FARM

Although lavage of mammalian lung recovers numerous respiratory macrophages [10], lavage of avian lungs is associated with failure to recover FARM [13]. However, under non inflammatory conditions, some studies report recovery of few FARM through repeated lavages of the parabronchial avian lungs [11, 12]. On average, the number of respiratory macrophages harvested by lavage of mammalian lungs exceeds the number of FARM recovered by similar method in captive birds by approximately 20 times. The average number of FARM in the rock dove is 1.6 × 10^5^ [9] while that in the domestic fowl and turkey is 2.5 × 10^5^ and 1.15 × 10^6^ respectively [10, 12]. Lavage of rat and hamster lungs yielded 8.5 × 10^6^ and 4.64 × 10^6^ respiratory macrophages respectively [10, 14]. An average of 1.1 × 10^6^ FARM and 1.5 × 10^7^ respiratory macrophages were harvested by lavage of domestic duck and rabbit respectively [15]. Paucity of FARM in avian lungs has been purported to foreordain a weak first line of defense against invading particulate matter and pathogens, a condition that has been used to explain high mortality among the captive birds to respiratory disease conditions [16].

## Survey of FARM in the avian lung

According to a review by [5], study of the avian cellular defenses processes is important for: (i) evaluation of drug delivery by aerosolization in a complex respiratory system, (ii) understanding the evolution of pulmonary cellular protection mechanisms and (iii) understanding the pathogenesis of respiratory disease conditions in birds. Microscopic techniques have been used to survey distribution of FARM in the avian lung. Numerous FARM have been localized in the atrial and infundibular subepithelium but the cells are absent in the air capillaries [17]. Morphologically, FARM are similar to mammalian alveolar macrophages exhibiting characteristic eccentric nucleus, a plasma membrane ruffled with filopodial extensions and lysosomes which appear as electron cytoplasmic vesicular bodies [15, 29].

## The role of FARM in avian pulmonary defense

According to [5], dearth of FARM in the avian respiratory system could imply: (a) a weak first line of defense of the avian respiratory system, (b) FARM population on the respiratory surface increases following transmigration of the cells from the avian lung subepithelium or vascular system in response to invading particulates and pathogens, (c) evolution of supplementary defenses, (d) FARM are efficiently cytotoxic to pathogens, or/and (e) FARM are quantitatively fewer but highly phagocytic. Existing data on the protective roles of FARM negate the assertion that dearth of the cells foreordains a weak first line of defense in the avian lung as outlined below.

### FARM transmigrate into avian lung in response to infections

Transmigration of FARM during repeated washings of avian lungs has been reported by [10, 12], a property that was investigated to examine the role of vaccines in enhancing FARM innate immunity in birds. Following intra-tracheal delivery of live but apathogenic *Pasteurella multocida* vaccine in chickens, [18] observed a threefold increase in the number of FARM harvested by lavage. However, the activated FARM showed pick of transmigration into the lung after 8 h post-inoculation and the FARM numbers reduced substantially after 3–4 days. The FARM were highly phagocytic and exhibited exceptional bactericidal activity. Further, chickens yielding a high number of FARM did not show any sign of respiratory disease, a concept [18] referred to as preventive activation.

### Pulmonary mechanisms supplementing protective roles of FARM

The ciliated epithelia lining trachea, primary bronchus and proximal portions of secondary bronchi supplement the FARM by removing particulates and pathogens through an efficient mucociliary escalator system enriched by mucus [19]. Further, the ciliated epithelial linings of the avian respiratory system have tight junctions offering a physical barrier against entry of microbes [20, 21].

Deposition of fine particulates in the avian system depicts an efficient three tiered aerodynamic filtration system. Detailed study by [22] showed that the deposition and clearance of particles on the avian airways is a function of particle size. Large particles (3.7–7 µm in diameter) are deposited and removed in the nasal cavities and proximal trachea. Midsize particles (1.1 µm) are trapped primarily in the lung and cranial air sacs while smaller particles (0.091 µm) pass through the entire lung and are finally trapped in abdominal air sacs.

Removal of small inert particles (non–toxic iron oxide aerosol, particle diameter 0.18 µm) from the lung was first investigated by [23] in a duck model. It was shown that these particles were not only phagocytosed by FARM but also by epithelial cells of the atria. These observations were subsequently confirmed in chickens, pigeons and ducks by [9]. From this work a picture emerges that epithelial cells and FARM play a crucial role in removal of particles from the air on their way to the thin, extensive and highly vulnerable tissue of the gas exchange area [24].

### FARM are cytotoxic to pathogens

The role of FARM in pathogenesis of aspergillosis was recently evaluated using *Aspergillus fumigatus* conidia. Pigeon FARM co-cultured with *A. fumigatus* conidia internalized and killed substantial amount of conidia. However, exposure of FARM to numerous conidia overwhelmed the ability the FARM to phagocytose and eliminate the spores. A small proportion of internalized conidia germinated in the FARM wit subsequent degeneration and necrosis of the macrophages [25]. Impairment of FARM function observed with *A. fumigatus* conidia overload has been reported in other works. Particle overload in macrophages, for instance, has been reported by [26] who concluded that macrophages can only engulf up to a given maximum volume of particles. Alveolar macrophage function has been reported to be impaired when an average 6 % of its volume is filled by phagocytosed particles [27], a suggestion that particle overload plays a role in the breaching of the pulmonary epithelia barrier.

### FARM are exceptionally phagocytic cells

Comparative in vitro studies on the phagocytic capacities of FARM and alveolar macrophages harvested by pulmonary lavage confirm FARM to have a significantly higher phagocytic index than the alveolar macrophages. In a study by [28], chicken FARM and rat alveolar macrophages were co-cultured with polystyrene particles and the subsequent analyzed volume density of internalized particles per unit volume of a cell was higher in the FARM. The volume density of phagocytized particles in the chicken FARM and rat alveolar macrophages was 23 and 5 % respectively [28]. The study further reported chicken erythrocytes recovered by lavage to be phagocytic; however, it remains to be shown whether this is an inherent property of avian nucleated erythrocytes. The FARM phagocytic index reported in this study was collaborated by a recent study in which chicken FARM co-cultured with polystyrene particles had a phagocytic capacity of 21 %, [29]. In a study by [15], FARM recovered by lavage of domestic duck lungs were reported to phagocytose more particles than alveolar macrophages harvested by similar method in the rabbits. The phagocytic index measured as a function of volume density of particles phagocytosed per unit volume of a duck FARM and a rabbit alveolar macrophage was 20 and 9 % respectively [15]. These experimental data support the assertion that FARM exhibit an exceptionally efficient phagocytic ability.

## Effects of PPAR γ ligands on FARM

Peroxisome proliferator activated receptors (PPAR) are transcription ligand activated nuclear receptors [30] and three isoforms: PPAR α, PPAR β and PPAR γ have been identified [31]. The PPAR γ protein is predominantly expressed in the adipose tissue [32]. Later the protein was also found to be expressed in immune cells including monocytes and macrophages [33]. Thiazolidinediones are selective synthetic PPAR γ ligands [34] and treatment of respiratory macrophages with high doses of selective synthetic PPAR γ ligands induces non phlogistic phagocytic properties in the cells with subsequent clearance of inflammatory sites in the mammalian lung [34]. Non phlogistic phagocytosis is characterized by enhanced ability to internalize particles and pathogens with attenuated production of proinflammatory cytokines by phagocytic cells [35].

Avian respiratory disorders are characterized by inflammation of the respiratory epithelium which is a complex biological response of lung and other tissues to harmful stimuli such as pathogens, damaged cells, or irritants such as reactive oxygen species [36]. Even though Inflammation is a protective attempt by an organism to remove the injurious stimuli as well as initiating the healing process for the tissue [37], prolonged inflammation has been associated with respiratory epithelial tissue destruction and pathogenesis of disease conditions such as aspergillosis in captive birds [38].

A review by [39] observes that “an understanding of the mechanisms and molecules that enhance FARM to regulate immune and inflammatory responses may permit the development of products, diets, or husbandry techniques to modulate immunity for the enhancement of the productivity of poultry”. Five specific rationales for modulating FARM function in poultry have been suggested by [39]. These are: (a) providing enhanced or sustained immune response to infectious organisms; (b) enhancement and direction of vaccination responses; (c) mitigation of immunosuppression arising from infectious diseases, dietary toxins, or stress; (d) accelerating the development and maturation of the immune system; (e) inducing tolerance to nonpathogenic environmental immunogens; and (f) mitigating the catabolic consequences of an immune response.

In a recent study that elucidated the anti-inflammatory roles of selective synthetic PPAR γ in FARM, chicken FARM were treated with high dose of troglitazone, a selective synthetic PPAR γ ligand. The study demonstrated that selective synthetic PPAR γ ligands improve the ability of freshly harvested FARM to internalize particles. The volume density of internalized particles per unit volume of a FARM was 41 and 21 % in treated and untreated FARM [29]. The study further observed that treatment of the FARM with PPAR γ ligands attenuated proinflammatory cytokine production in activated FARM [29]. These data indicate that synthetic PPAR γ ligands could be used to improve the ability of FARM to resolve inflammatory disease condition through non phlogistic clearance of inflammatory sites in the avian lung.

## Conclusions

Paucity of FARM in the avian lung may not necessarily imply a weak first line of pulmonary defense. FARM, with exceptional bactericidal ability, transmigrate into avian lung in response to infections. An efficient aerodynamic filtration system, tight epithelial junctions that provide a physical barrier against invading pathogens and ciliated epithelium in the trachea and bronchi which removes foreign substances through an efficient mucociliary escalator system, supplement the protective roles of FARM. Available experimental data confirm FARM to have exceptionally efficient phagocytic ability. Recently, selective synthetic PPAR γ ligands have been shown to induce non phlogistic phagocytic properties in the FARM, a necessary condition for resolution of inflammatory disease conditions in the lungs. To develop effective therapeutic interventions targeting FARM in treatment and management of respiratory disease conditions in the poultry, further studies are required to fully understand the role of FARM in innate and adaptive immune responses.
